# A three-dimensional organoid model recapitulates tumorigenic aspects and drug responses of advanced human retinoblastoma

**DOI:** 10.1038/s41598-018-34037-y

**Published:** 2018-10-23

**Authors:** Duangporn Saengwimol, Duangnate Rojanaporn, Vijender Chaitankar, Pamorn Chittavanich, Rangsima Aroonroch, Tatpong Boontawon, Weerin Thammachote, Natini Jinawath, Suradej Hongeng, Rossukon Kaewkhaw

**Affiliations:** 1 Research Center, Faculty of Medicine Ramathibodi Hospital, Mahidol University, Bangkok, Thailand; 2 Department of Ophthalmology, Faculty of Medicine Ramathibodi Hospital, Mahidol University, Bangkok, Thailand; 3 Bioinformatics Computational Biology Core, National Heart, Lung, and Blood Institute (NHLBI), National Institutes of Health (NIH), Bethesda, USA; 4 Section for Translational Medicine, Faculty of Medicine Ramathibodi Hospital, Mahidol University, Bangkok, Thailand; 5 Department of Pathology, Faculty of Medicine Ramathibodi Hospital, Mahidol University, Bangkok, Thailand; 6 Department of Pediatrics, Faculty of Medicine Ramathibodi Hospital, Mahidol University, Bangkok, Thailand

## Abstract

Persistent or recurrent retinoblastoma (RB) is associated with the presence of vitreous or/and subretinal seeds in advanced RB and represents a major cause of therapeutic failure. This necessitates the development of novel therapies and thus requires a model of advanced RB for testing candidate therapeutics. To this aim, we established and characterized a three-dimensional, self-organizing organoid model derived from chemotherapy-naïve tumors. The responses of organoids to drugs were determined and compared to relate organoid model to advanced RB, in terms of drug sensitivities. We found that organoids had histological features resembling retinal tumors and seeds and retained DNA copy-number alterations as well as gene and protein expression of the parental tissue. Cone signal circuitry (M/L^+^ cells) and glial tumor microenvironment (GFAP^+^ cells) were primarily present in organoids. Topotecan alone or the combined drug regimen of topotecan and melphalan effectively targeted proliferative tumor cones (RXRγ^+^ Ki67^+^) in organoids after 24-h drug exposure, blocking mitotic entry. In contrast, methotrexate showed the least efficacy against tumor cells. The drug responses of organoids were consistent with those of tumor cells in advanced disease. Patient-derived organoids enable the creation of a faithful model to use in examining novel therapeutics for RB.

## Introduction

Retinoblastoma (RB) is a serious childhood retinal tumor that, if left untreated, can cause death within 1–2 years. Current management of RB aims to salvage both the globe and visual function, in addition to saving the patient’s life. Management of advanced RB involves attention to tumors at three anatomical sites, including the individual retinal tumor (s), associated vitreous tumors and associated subretinal tumors (termed “vitreous seeds” and “subretinal seeds”)^1^. Presence of seeds in advanced RB is associated with disease recurrence, which is a major cause of chemoreduction failure and represents the primary limitation for globe salvage^1,2^. Systemic intravenous chemotherapy encounters difficulty in controlling the seeds that exhibit massive and diffuse infiltration^1,3^; the minimal response to chemotherapy is partly because of resistance of the tumor seeds to chemotherapy^3–6^ or avascular sites in the vitreous cavity and subretinal space, causing inadequate penetration of delivered drugs.

In addition to primary treatment intravitreal chemotherapy is locally applied to increase drug accessibility and shows impressive control of vitreous seeds^3–7^ as well as subretinal seeds and recurrent retinal tumors^8,9^ with minimal complications. Melphalan is extensively used despite its high toxicity^4,5^: this therapy results in an overall globe salvage rate of 68%^3,7^. A few drugs, such as topotecan and methotrexate, have been used with variable degrees of success^6,10^; the combination of topotecan and melphalan is optional for refractory and recurrent seeds or retinal tumors^8,9,11^. However, case reports have shown failure in some patients, leading to enucleation^3–7,10^. This highlights the need for drug development and evaluation to ascertain efficacy and safety. Representative and robust models of advance RB are thus required to determine the activities of candidate therapeutic agents for control of retinal tumors and seeds.

Genetically engineered mouse models (GEMMs) are powerful tools to study pathogenesis and develop new therapies for RB^12,13^. Unlike in human RB, additional genes must be inactivated together with *Rb1* to induce tumorigenesis in mice^13–15^. Molecular and cellular analyses indicate that mouse RB has properties of amacrine/horizontal interneurons, reflective of the tumor cells of origin^12,14,16^. In contrast, cones are frequently identified in human RB^17^ and significantly sensitive to cancerous transformation when the *RB1* gene is lost in the human retina^18^. Furthermore, the epigenetic landscape significantly differs between mouse and human RB^16,19^. Some candidates for molecular targeted therapy, such as epigenetically deregulated *SYK*^20^ in human RB, appear to be normally regulated in GEMMs^19^. This indicates that different mechanisms underlying tumorigenesis exist between humans and mice.

Advances in organoid technology allow the generation of three-dimensional (3-D), self-organizing tissue that encompasses multiple lineages through a nature-mimicking process. Accordingly, human and murine organoids have been generated from pluripotent or tissue stem cells in both healthy and diseased conditions^21^ and then used to facilitate better understanding in biology and pathology^22–25^. Solid tumor tissues from patients have been used to generate organoids that retain molecular and histopathologic features of the original primary tumor tissue. This has been demonstrated in colon^26,27^, breast^28^, liver^29^, prostate^30^, and pancreatic tumors^31^, but has not yet been demonstrated for retinal tumors. Here, we aim to establish a model of advanced RB through organoid culture derived from RB tissues for drug testing. Cellular and molecular features are thoroughly characterized to ascertain the presentation of tumorigenic aspects of the parental tumors in organoids after short and long-term culture. As a proof-of-concept for modeling advanced RB, we determine and compare the responses of tumor organoids to clinically used drugs for intravitreal chemotherapy^3–11^ to relate organoid model to advanced RB, in term of drug sensitivities. We further demonstrate that drugs with greater efficacy not only induce cell death, but also preferentially target proliferative tumor cones, rather than resting cones. Thus, organoids provide opportunities for drug testing and the development of targeted therapies for RB.

## Results

### Establishment of expansible RB organoids

**Figure 1:**
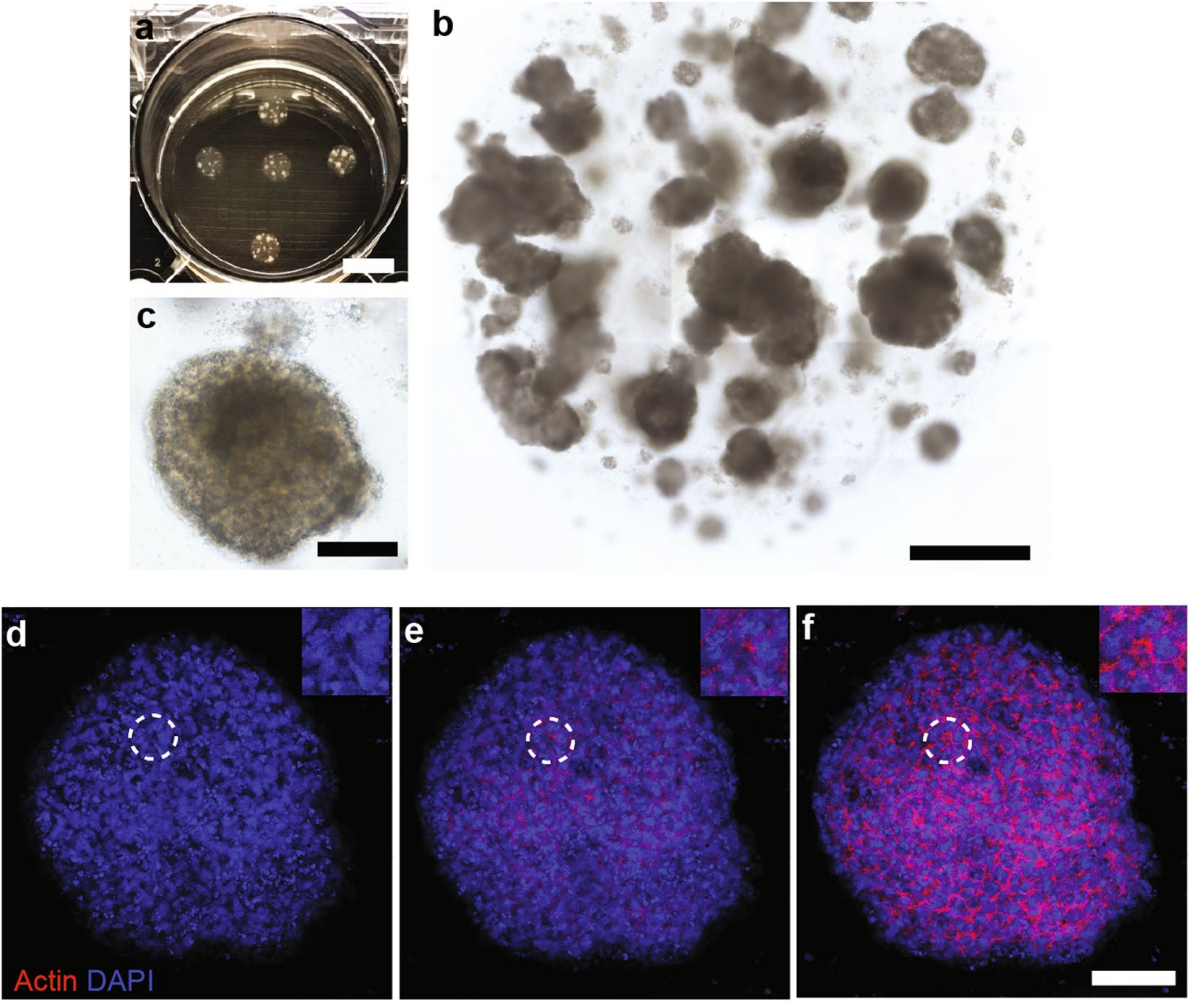
Establishment of retinoblastoma organoid culture. (**a**) Photograph of retinoblastoma organoids grown in Matrigel® drops. (**b**) Mosaic image shows multiple organoid sizes in a single Matrigel® drop; typical growth features of a 3-week culture after passaging. (**c**) Magnified micrograph of organoid showing dense cellular organization. (**d**–**f**) Confocal z-plane images of whole-mount organoid (bottom to top), stained with phalloidin and 4′,6-diamidino-2-phenylindole (DAPI), showing multiple rosette formation (dashed-line circles indicate inserted images). Scale bar, 1 cm (**a**); 1000 µm (**b**); 200 µm (**c**) and 100 µm (**d**–**f**).

Fresh surgical specimens of chemotherapy-naïve RB were obtained and processed for organoid derivation (∼0.3 cm^3^ tissue), as well as genomic and transcriptomic analyses. Tissue was mechanically and enzymatically dissociated; dissociated cells were mixed in with Matrigel® solution and plated as adherent Matrigel® drops which were overlaid with culture medium. We initially attempted to grow tumor organoids in medium (insulin, transferrin, N2 supplement, and FBS) for retinal organoids derived from pluripotent stem cells^22^, which failed to support the growth. We then used mitogens (EGF and FGF2, known to support the survival of retinal cells^32^), serum replacement, and culture medium supporting the growth of neural progenitors. This newly formulated medium supported the proliferation of patient-derived cells that previously failed to grow (data not shown). Hence, we used newly formulated medium, in combination with Matrigel®, to establish tumor organoid cultures from the RB tissues of a new patient. This method efficiently allowed generation of tumor organoids and long-term expansion (>8 passages). A cluster of cells initially formed in Matrigel®, then enlarged and became dense (Fig. 1a−c). Organoids were present in multiple sizes up to 1 mm in each single drop of Matrigel® at 3 weeks post-seeding; the cultures could be serially expanded with a consistent passaging ratio of 1:3–1:4 (Fig. 1a,b). Individual organoids displayed dense cellular organization of elements resembling rosette formation (Fig. 1c–f).

Tumor tissues were obtained from six enucleated eyes, classified into group E (International Classification of Retinoblastoma); all globes contained retinal tumor (s) and associated vitreous or/and subretinal seeds (Table 1). Unlike in tumor seeds^33^, retinal tumors contained a great number of viable tumor cells and were thus used for organoid culture. Five (RB688, RB654, RB187, RB183, and RB521) out of six organoid lines were successfully established, demonstrating 83% success rate of organoid derivation from retinal tumors (Table 1). Furthermore, RB organoids could be stored and resurrected from long-term storage in liquid nitrogen (up to 5 months’ storage was tested for RB688) and retained normal cellular structure (data not shown).

**Table 1:** Case characteristics and features of retinal tumors and seeds in enucleated globes.

Case*	Age at diagnosis (months)	Laterality	Differentiation of primary retinal tumors	Presence of seeds**
RB668	7	Bi	Well-differentiated	Subretinal
RB654	3	Bi	Well-differentiated	Vitreous (sphere)
RB187	29	Uni	Poorly differentiated	Subretinal and vitreous (cloud, sphere)
RB183	3	Bi	Moderately differentiated	Vitreous (sphere, dust, cloud)
RB521	22	Uni	Moderately differentiated	Vitreous (dust, sphere)
RB261	27	Uni	Moderately differentiated	Vitreous (dust, sphere)

*All eyes are classified into group E according to International Classification of Retinoblastoma; all cases undergo primary enucleation. **Types of vitreous seeds are classified with clinicopathologic examination; subretinal seeds are identified with histopathological analysis. Abbreviations: Uni, unilateral RB; Bi, Bilateral RB.

### RB organoids maintain cellular features of parental tumor

**Figure 2:**
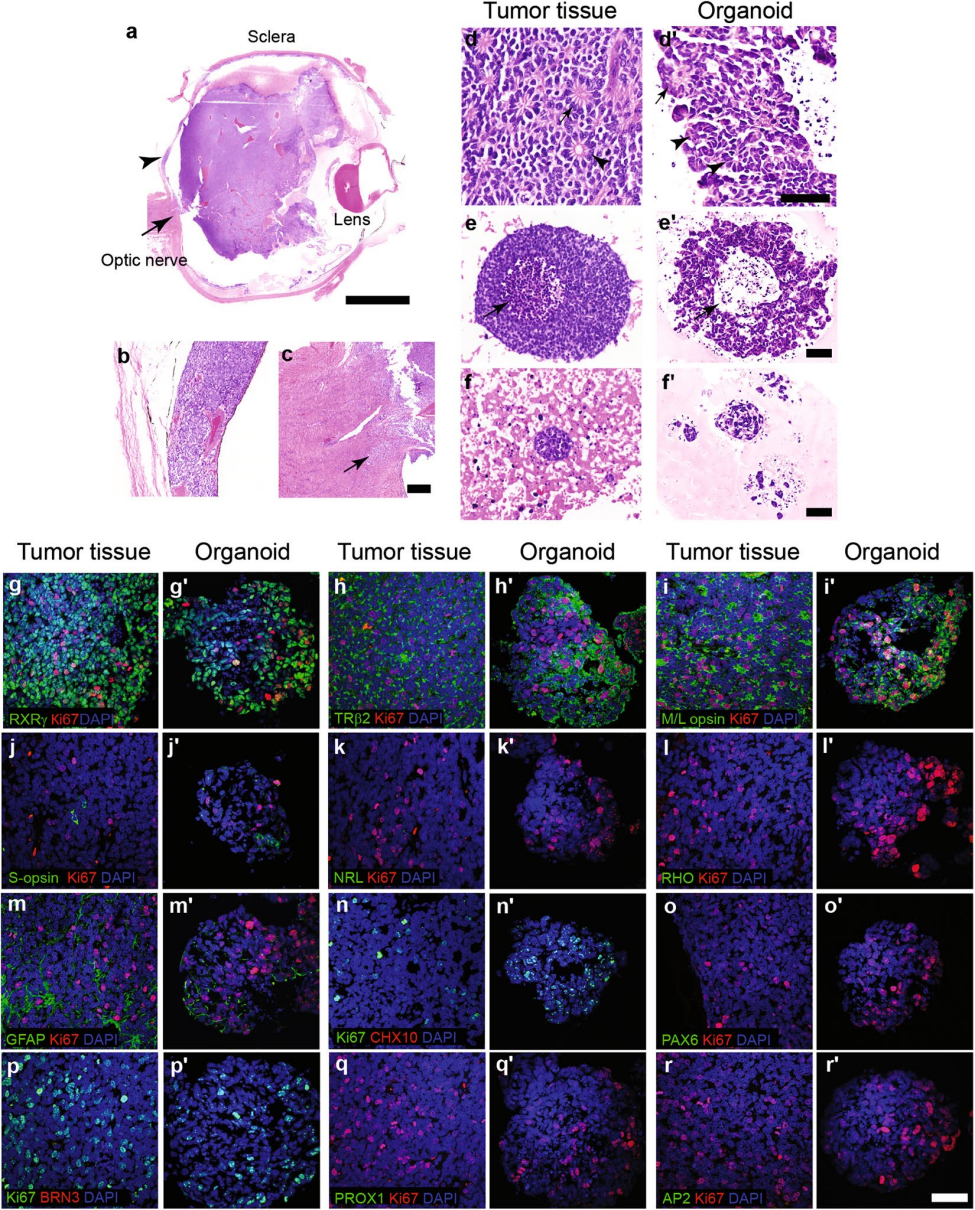
Reproducible cellular features and contents of the retinoblastoma in tumor organoids. (**a–c**) Hematoxylin and eosin staining of the enucleated globe (**a**). Arrow head and arrow in (**a**) indicate magnified regions showing choroid (**b**) and optic nerve (c, (arrow)) invasion, respectively. (**d–f’**) Representative micrographs indicate histological features of retinal tumor (**d**) and subretinal seeds (**e**,**f**) in tumor tissue and the corresponding structures in organoids (RB668) (**d’**,**e’**,**‘f**). Flexner-Wintersteiner (arrowhead), Homer-Wright (arrow) rosettes (**d**,**d’**) and features of tumor seeds, including a large spherical cluster with an outer rim of viable cells surrounding central apoptotic cells (arrow) (**e**,**e’**) and a small spherical cluster of viable cells (**f**,**f’**) identified in tissue and cultures. (**g–r’**) Representative micrographs of co-immunostaining indicate the expression of Ki67 and retinal proteins [RXRγ (**g**,**g**’), TRβ2 (staining specificity demonstrated by Xu *et al*.^17^) (**h**,**h’**), M/L opsin (**i**,**i’**), S- opsin (**j**,**j’**), NRL (**k**,**k’**), RHO (**l**,**l’**), GFAP (**m**,**m’**), CHX10 (**n**,**n’**), PAX6 (**o**,**o’**), BRN3 (**p**,**p’**), PROX1 (**q**,**q’**), and AP2 (**r**,**r’**)] in parental tumor tissue and organoids. Nuclei stained by 4′,6-diamidino-2-phenylindole (DAPI). Scale bar, 5 mm (**a**); 200 µm (**b**,**c**); 100 µm (**d**,**d’**) and 50 µm (**e–f’** and **g–o**). See Figs S1 and S2 for other organoid lines (RB654 and RB187).

Histological analysis revealed that RB filled almost the entire globe and displayed massive choroidal and laminar optic nerve invasion (Figs 2a–c, S1a and S2a for RB668, RB654 and RB187, respectively). The parental RB demonstrated cuboidal cells with hyperchromatic nuclei and scant cytoplasm; this morphology was also found in tumor organoids (Figs 2d,d’, S1b,b’ and S2b,b’). Histological features of parental tumor tissues, including the formation of Flexner-Wintersteiner and Homer-Wright rosettes, were identified in RB668 organoids (Fig. 2d,d’). In addition, cellular structures corresponding to subretinal seeds were identified in organoids; these included large spherical clusters with an outer rim of viable cells surrounding central apoptotic cells (Fig. 2e,e’) and small spherical clusters of viable cells (Fig. 2f,f’). Similarly, histological features of retinal tumors and tumor seeds in the parental tissues were reproducible in RB654 and RB187 organoids (Table 1 and Figs S1b−c’ and S2b−c’). Dust, sphere and cloud types of vitreous seeds have been classified; spheres contain the greatest proportion of viable tumor cells^33^. We found that sphere type-like seeds were often present in organoids (Table 1 and Figs S1c’ and S2c’). Altogether, organoids retained the histological features of retinal tumors and seeds and thus represented a model of advanced RB.

To determine cellular phenotypes, retinal cell and Ki67-proliferative markers were co-labeled in tumor organoids (RB668, RB654 and RB187) and the corresponding patient-derived tissues (Figs 2g–r’, S1d−o’ and S2d−o’). This co-labeling enabled identification of a specific type of retinal tumor cell, which had the capability of neoplastic growth. Immunostaining revealed that retinoid X receptor-γ (RXRγ) and thyroid hormone receptor β2 (TRβ2), transcription factors important for the differentiation and maintenance of M/L cone identity^34,35^, were detected in a majority of tumor cells within tissues and organoids (Figs 2g–h’, S1d−e’ and S2d−e’). A subset of RXRγ^+^ and TRβ2^+^ cells was co-labeled with Ki67 (Figs 2g–h’, S1d−e’ and S2d−e’). Detection of M/L opsin^+^ cells and M/L opsin^+^ Ki67^+^ cells confirmed the presence of neoplastic M/L cones in tumor tissues and organoids (Figs 2i,i’, S1f,f’ and S2f,f’). In contrast, S opsin^+^ cells were rarely detected and did not express Ki67 (Figs 2j,j’, S1g,g’ and S2g,g’), suggesting that S opsin^+^ cells are non-proliferative. The expression of rod cell markers (neural retina-specific leucine zipper protein (NRL) and rhodopsin) was not detected in organoids and parental tumor tissues (Figs 2k–i’, S1h−i’ and S2h−i’). In addition to photoreceptors, we examined organoids and their corresponding RB tissues for the expression of other retinal proteins. Glial fibrillary acidic protein (GFAP)^+^ Ki67^−^ cells were detected, suggesting the presence of non-proliferative glial cells in tumor organoids, similar to parental tumor tissues (Figs 2m,m’, S1j, j’ and S2j, j’). In contrast, retinal progenitor (CHX10 and PAX6), ganglion (BRN3 and PAX6), bipolar (CHX10), amacrine (PROX1, AP2-α, and PAX6), and horizontal (PROX1 and PAX6) cells were absent in organoids as indicated by undetectable marker proteins, a finding that concurred with data from tumor tissues (Figs 2n–r’, S1k−o’ and S2k−o’). Altogether, the results demonstrated that RB organoids recapitulated and retained retinal protein expression of the parental tumor tissues. Detailed analysis also indicated that neoplastic cells retained M/L cone phenotypes, even after long-term expansion in culture or storage in liquid nitrogen, in the same culture conditions (data not shown).

### RB organoids retain genetic alterations of original tumor tissue

**Figure 3:**
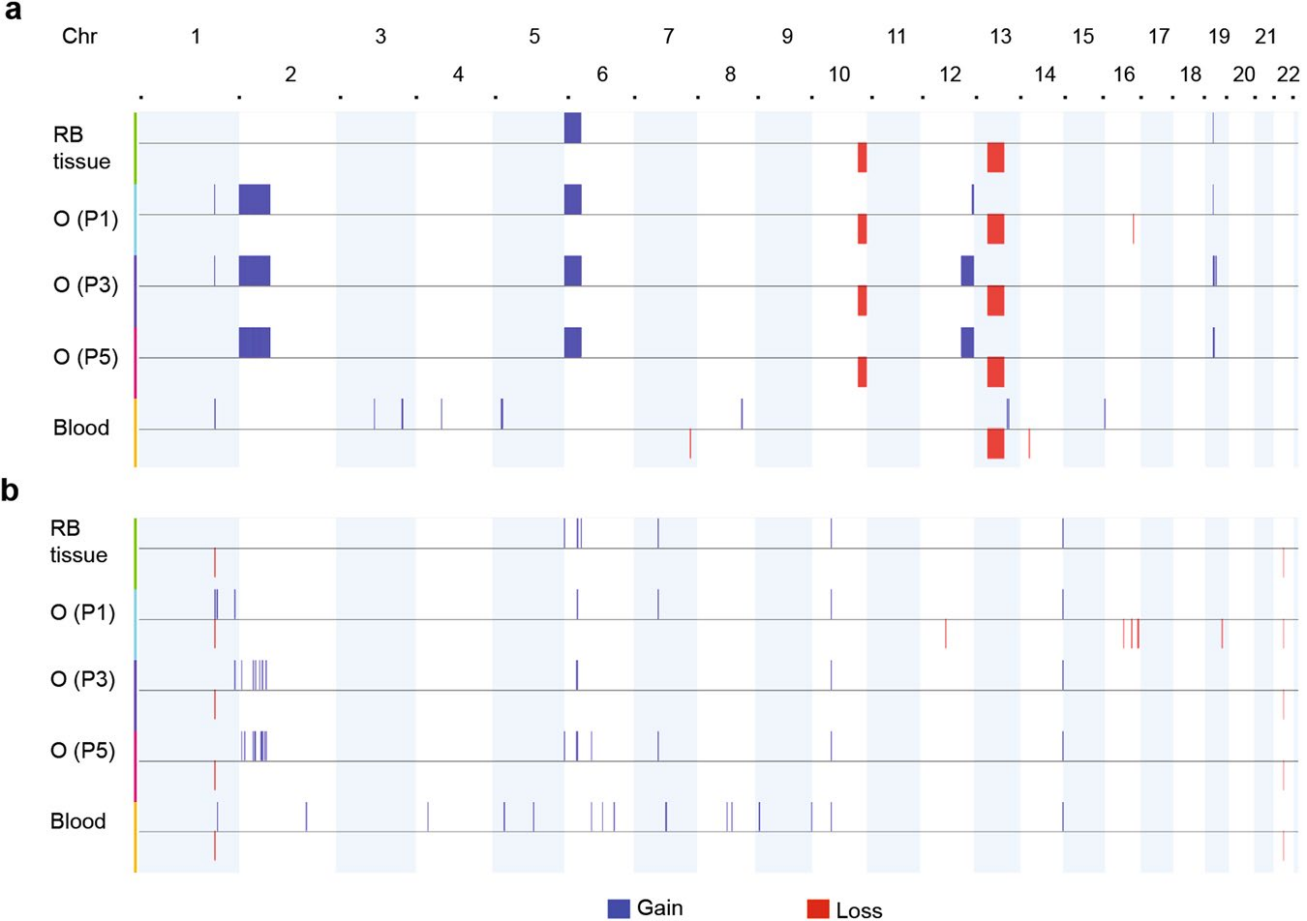
DNA copy number landscape of patient-derived retinoblastoma organoid line. (**a**,**b**) Copy number aberration of regional gains and losses (>3 Mb) (**a**) and focal lesions (<3 Mb) (**b**) in retinoblastoma (RB) tissue, RB688 organoids (O) at passage 1 (P1, 6-week culture), 3 (P3, 13-week culture), and 5 (P5, 19-week culture), matched with peripheral blood. See Fig. S3 for the frequency of gains or losses in tissue and organoids.

While the initiation of RB occurs as a result of *RB1* biallelic loss, recurrent genomic gains and losses drive tumor progression. These alterations were determined in organoid cultures (RB668) at 6 (P1), 13 (P2), and 19 (P5) weeks, in comparisons of tumor tissue matched with peripheral blood. Screening for *RB1* mutations identified a large deletion (13q13.1–13q22.2) spanning the *RB1* gene (Fig. 3a) as a germline mutation. An additional mutation (g.41924 A > G) caused defective splicing of *RB1* transcripts in the retinal cells that became malignant transformation. The biallelic loss of *RB1* was present in patient-derived organoids (Fig. 3a). The recurrent regional gains (>3 Mb) were consistently identified at 6p25.3–6p21.1 and 19p12–19p11; losses occurred at 10q25.2–10q26.3 in parental tumor and organoids at different serial passages (Fig. 3a). In addition, recurrent copy number aberrations were frequently found in tumor organoid cells, indicating that sub-clonal populations found in tumor tissue were enriched in organoids; this was consistently maintained with serial passaging (Figs 3 and S3a,b).

Two additional large regional gains (2p25.3–2p12 and 12q23.3–12q24.33) were identified in organoids (Figs 3a and S3c,d); sub-clonal populations with these gains were further enriched with serial passaging (Fig. S3c,d). In addition, focal lesions (<3 Mb) were detected within the same fragments, with large regional gains consistently identified at chromosomes 2 and 6 and inconsistently identified at chromosome 16 (Fig. 3b). Somatic copy number alterations, including 1q, 2p, and 6p gains, as well as 16q loss, are commonly identified in RB^36,37^. In addition, the recurrent 6p gain is associated with 2p gain, while the 1q gain is associated with 16q loss; the former association precedes the latter and thus is identified in RB tumors from patients diagnosed at younger age^38^. This suggests that 2p gain could be expected in organoid cells that were derived from the tumor with the recurrent 6p gain in our young patient at 7 months of age at diagnosis. Loss of heterozygosity was consistently maintained between tissue and organoids at different passages (data not shown).

### Gene expression profile reflects the origin of RB in tumor organoids

**Figure 4:**
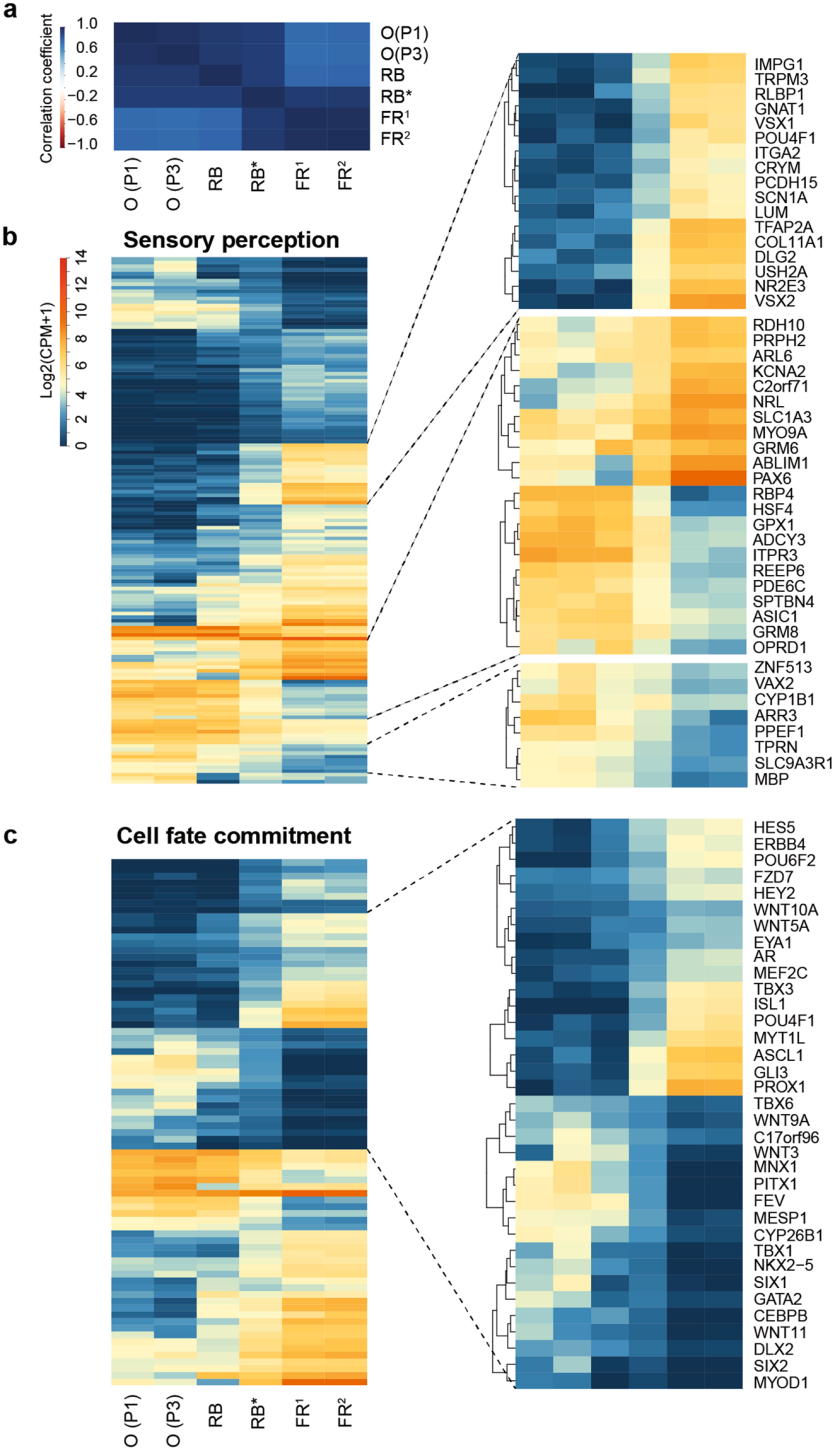
Tumor organoids recapitulate gene expression profile of primary retinoblastoma tissue. (**a**) Correlation matrix heat map between RB688 organoids (O) at passage 1 (P1, 6-week culture) and 3 (P3, 13-week culture), the corresponding patient-derived retinoblastoma (RB), and published transcriptomes of retinoblastoma (RB^*^) and fetal retina (FR^1^ and FR^2^). (**b**,**c**) heat maps show expression levels of genes associated with the top two significantly enriched gene ontology (GO) terms: sensory perception (**b**) and cell fate commitment (**c**). See Fig. S5 for other GO terms and the expression levels of associated genes.

Gene expression profiling from RNA-seq data was conducted to determine whether tumor organoids (RB668) retain a gene signature of the parental tumor. Since the tumor was diagnosed at early age (7 months) in our RB patient, we included published transcriptome data of fetal retina (19 weeks)^39^ and RB^16^ for analysis (Figs 4, S4–S6). Gene profiling analysis revealed that tumor organoids strongly correlated with the parental tumor and were consistent between passages (Fig. 4a). As expected, the organoids and tumor tissue had a higher degree of correlation with primary RB than with normal developing retina (Fig. 4a). Furthermore, gene expression profiles of our samples were more readily distinguishable from normal retina (Fig. 4a) than in reported cases of RB, suggesting a higher purity of tumor cellularity in our samples. Tumor organoids and the patient’s tumor had high expression levels of cone-enriched genes, consistent with the analysis of protein expression (Figs 2g−r’ and S4a). In addition, cone-associated genes that are susceptible to RB transformation were enriched in tumor and organoids, in response to *RB1* inactivation (Fig. S4b).

Differential expression analysis of tumor organoids and fetal retina revealed 2723 genes; functional annotation of differentially expressed genes (DEGs) was conducted to assist in identifying histogenesis of organoids. Gene ontology (GO) analysis revealed that sensory perception was the most significantly enriched GO term (Figs 4b, S5a). We found that genes with depleted levels of expression in organoids (and tumor) were associated with the development and function of retinal neurons [ganglion (*POU4F1* (*BRN3A*), *KCNA2*, and *SCN1A*), horizontal and amacrine (*TFAP2A* (*AP2-α*) and *PAX6*), and bipolar (*VSX1* and *GRM6*) cells], Müller glial (*RLBP1* and *SCL1A3*), and retinal progenitor (*VSX2* and *PAX6*) cells (Fig. 4b). In addition, the expression levels of rod-enriched genes, including *NRL*, *NR2E3*, *CNGA1*, and *PDE6G*, were depleted in organoids, consistent with tumor, but high in normal retina, where rods outnumber cones (Fig. 4b). In contrast, the expression levels of cone-enriched genes (*PDE6C* and *ARR3*) were high in tumor and further enriched in organoids, compared with fetal retina (Fig. 4b).

**Figure 5:**
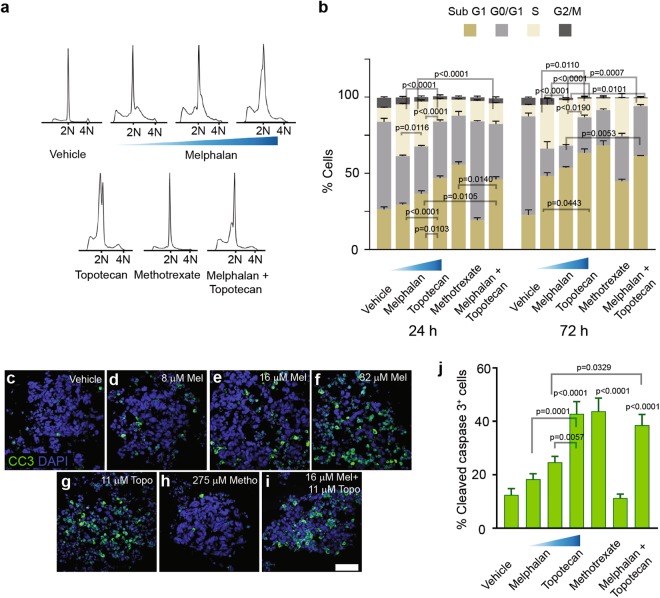
Chemotherapeutic drug responses of tumor organoids. (**a**,**b**) Cell cycle analysis of RB688 organoids in response to anticancer drugs at 24 (**a**,**b**) and 72 (**b**) h after drug administration. Statistical analysis of cell cycle phases at each time point (mean percentage ± SEM, n = 3) was conducted by one-way ANOVA followed by Tukey’s test. (**c–i**) Representative micrographs of immunostaining for cleaved caspase 3 (CC3), an indicative marker of apoptotic cells, in organoids treated with vehicle (**c**), 8 (**d**), 16 (**e**), or 32 (**f**) µM melphalan (Mel), 11 µM topotecan (Topo) (**g**), 275 µM methotrexate (Metho) (**h**), or the combined regimen of 16 µM melphalan with 11 µM topotecan (**i**). Nuclei stained by 4′,6-diamidino-2-phenylindole (DAPI). Scale bar, 50 µm. (**j**) Bar graph indicates % CC3^+^ cells (mean percentage ± SEM, n = 3) after exposure to drugs for 24 h. Mean percentages were determined from 7–10 micrographs containing 300–500 cells for each condition. Statistical analysis of % CC3^+^ cells was conducted by one-way ANOVA followed by Tukey’s test. The p values of single or combined agents vs. vehicle for cell cycle analysis are listed in the text.

Cell fate commitment was enriched as the second most significant GO term (Figs 4c, S5a). The expression levels of cell fate regulatory genes in neuroretinal lineages were depleted in organoids (and tumor), compared with fetal retina. These included early expressed genes in retinal development (*TBX3*, *PAX6*, *NR2E1*, *EYA1*, and *GLI3*) and regulatory genes for maintaining the retinal progenitor program (Notch signaling: *HES5* and *HEY2*). Similarly, depleted expression levels were detected in organoids for genes directing neurogenesis (*ASCL1* and *MYT1*) and the formation of more specific retinal cell types [horizontal and amacrine (*PROX1*), ganglion (*ISL1*, *POU4F1*, and *POU6F2*), and rod (*MEF2C*) cells] (Fig. 4c). However, the expression levels of genes governing mesodermal cell lineage (*TBX6*, *WNT11*, *PITX1*, *FEV,* and *CYP26B1*) and functioning in the specification of mesodermal cells (*MESP1*, *TBX1*, *NKX2*.*5*, *SIX1*, *SIX2*, *GATA2*, and *MYOD1*) were enriched in organoids (and tumor), compared with fetal retina (Fig. 4c). Furthermore, the expression levels of tumor invasion-associated genes (*MMP17* and *ITGA3*) were enriched in organoids (and tumor) (Fig. S5a,b). A similar phenomenon was observed for the expression level of *SYK*, contributing to tumor progression after *RB1* inactivation^20^ (Fig. S5a,c). In addition, fibroblast proliferation and mesenchymal cell proliferation were significantly enriched GO terms; the expression levels of *TGFβ1*, *S100A6*, *PDGFA*, and *BMP7* were enriched in organoids (and tumor), compared with fetal retina (Fig. 5Sd,e). Altogether, this suggested that organoids (and tumor) contained hybrid gene signatures for both cone and mesodermal cells.

In addition, differential expression analysis revealed a smaller number of genes in organoids and tumor, compared with organoids and fetal retina (649 gene versus 2723 genes). We determined GO terms that were enriched in the set of 649 DEGs, assisting in identifying the main differences between organoids and tumor. The significantly enriched GO terms included blood vessel development (*VEGFA*, *PRDM1*, *EPAS1*, and *PECAM1*) and extracellular matrix/structure organization (*COL4A5*, *MFAP4*, *FN1*, and *FBLN2*) (Fig. S6a,b). Tumor tissue and retinal tissue had a similar expression pattern of genes associated with these GO terms, compared with organoids; the expression levels of most genes were depleted in organoids, agreeing with less complexity of biological features of organoids relative to the tissues (Fig. S6a,b).

### RB organoids allow *in vitro* evaluation of anticancer activity of drugs for RB control

To determine whether drug responses of advanced RB are reproduced in organoids cultures (RB688) were treated with clinically used drugs for intravitreal chemotherapy (melphalan, topotecan, and methotrexate). Furthermore, comparisons were made between combined drug (melphalan and topotecan) and single drug regimens, which are challenging to systematically perform in clinics. Concentrations of drugs used in this study were equivalent to the final clinical dose achieved in the vitreous cavity. Since tumor organoids exhibited cellular structure similar to tumor tissue (Fig. 1d–f), we demonstrated that drug accessibility and uptake occurred in the deepest area at the core of tumor organoids, indicated by elevated γ-H2AX foci, a DNA damage response marker (Fig. S7).

Cell cycle profiles (Fig. 5a,b) and apoptosis (Fig. 5c–j) were determined in response to anticancer drugs for short (24 h) and long (72 h) exposure times. Melphalan, a common clinical therapy for seed control, was examined at different doses. Melphalan at 8 µM significantly reduced the number of G0/G1-phase cells (p < 0.0001) and induced S-phase arrest (p < 0.0001) (Fig. 5a,b). However, this concentration was not sufficient to cause significant cell death, as there was no alteration in the number of sub-G1 and CC3^+^ cells in treated organoids (Fig. 5a–d,j). Higher concentrations of melphalan (16 and 32 µM) significantly induced elevated sub-G1 fractions (vs. vehicle, p = 0.0048 and p < 0.0001) (Fig. 5a,b), consistent with CC3^+^ staining for 32 µM melphalan (vs. vehicle, p < 0.001) (Fig. 5c,e,f,j). Elevated sub-G1 correlated with reduction of G0/G1 fractions (vs. vehicle, p < 0.0001) for both 16 and 32 µM concentrations of melphalan. The effect was more deleterious for the highest dose, reducing the G2/M-phase fraction (vs. vehicle, p = 0.0277) (Fig. 5a,b).

Unlike 8 and 16 µM melphalan, tumor organoid cells treated with 32 µM melphalan did not arrest in S phase, but underwent apoptosis in sub-G1 phase (8 vs. 32 µM, p < 0.0001; 16 vs. 32 µM, p = 0.0103) (Fig. 5a,b), consistent with CC3^+^ staining (8 vs. 32 µM, p = 0.0001; 16 vs. 32 µM, p = 0.0057) (Fig. 5j). This suggested that after 24 h of exposure, 8 and 16 µM melphalan preferentially induced S-phase arrest; in contrast, 32 µM melphalan immediately targeted organoid cells. When drug exposure time was prolonged to 72 h, melphalan at all doses significantly increased sub-G1 fractions (vehicle vs. 8 µM, p = 0.0006; vehicle vs. 16 µM, p < 0.0001; vehicle vs. 32 µM, p < 0.0001) and concomitantly reduced G0/G1 fractions (vehicle vs. 8, 16, 32 µM; p < 0.0001) (Fig. 5b). Treatment with 8 and 16 µM melphalan induced S-phase arrest (vehicle vs. 8 µM, p = 0.0029; vehicle vs. 16 µM, p < 0.0047), which was similar to 24 h exposure, but was sufficient to stop G2/M-phase entry [vehicle vs. 8 µM, p = 0.0045; vehicle vs. 16 µM, p < 0.0001) (Fig. 5b). This indicated that melphalan at low doses required a longer exposure time for anticancer activities.

Topotecan at 11 µM demonstrated efficiently reduced the number of tumor cells in G0/G1 and G2/M phases (vs. vehicle, p < 0.0001 and p = 0.0237) and simultaneously induced sub-G1 phase (p < 0.0001) in treated organoids, consistent with the elevated number of CC3^+^ cells (p < 0.0001) (Fig. 5a–c,g,j). Similar results regarding cell cycle distribution were obtained at 72 h of exposure, while further prolonging the incubation period increased cell death and reduced the number of G0/G1-phase cells (Fig. 5a,b). In addition, topotecan and the highest doses of melphalan showed similar cell cycle profiles (Fig. 5b), resulting in comparable killing effects in treated organoids (Fig. 5c,f,g,j).

Methotrexate induced S-phase arrest and subsequently prevented G2/M-phase entry (vs. vehicle, p = 0.0234 and p = 0.0465) (Fig. 5a,b). However, similar to 8 µM melphalan, the drug was not sufficient to substantially induce cell death at 24 h of exposure, consistent with CC3^+^ staining (Fig. 5a–d,h,j). Prolonged exposure to methotrexate simultaneously caused a reduction the number of G0/G1-phase cells and increased cell death in sub-G1 phase (vs. vehicle, p < 0.0001 and p = 0.0031) while maintaining action in S and G2/M phases (Fig. 5b). This indicated that methotrexate had a slow anticancer effect.

To increase efficiency in controlling tumor growth, combined melphalan and topotecan is used clinically^11^, but the comparative genotoxic effect of combinatorial drugs, relative to each single drug, has been unknown. Hence, 16 µM melphalan and 11 µM topotecan were tested in tumor organoids. The combined drug regimen significantly reduced S-phase arrest relative to that induced by melphalan alone (p < 0.0001), in concert with increased cell death in sub-G1 phase (p = 0.0105) (Fig. 5a,b); this was consistent with an elevated number of CC3^+^ cells (p = 0.0329) (Fig. 5e,g,i,j). Cell cycle distribution was generally similar to topotecan alone (Fig. 5b). Prolonged exposure to the combined drug regimen caused an increased G0/G1 fraction, relative to that induced by either agent alone, indicative of cell arrest (vs. melphalan, p = 0.0053) (Fig. 5b). This subsequently prevented S- and G2/M-phase entry in a significantly greater proportion of cells than melphalan alone (p = 0.0007 and p = 0.0101) (Fig. 5b). Altogether, this suggested that the genotoxic effect of the combined drug regimen was superior to melphalan alone; however, the combined drug regimen and topotecan alone appeared to have comparable effects in terms of cell cycle distribution and CC3^+^ staining.

### Combined treatment with melphalan and topotecan effectively targets neoplastic cone cells in organoids

**Figure 6:**
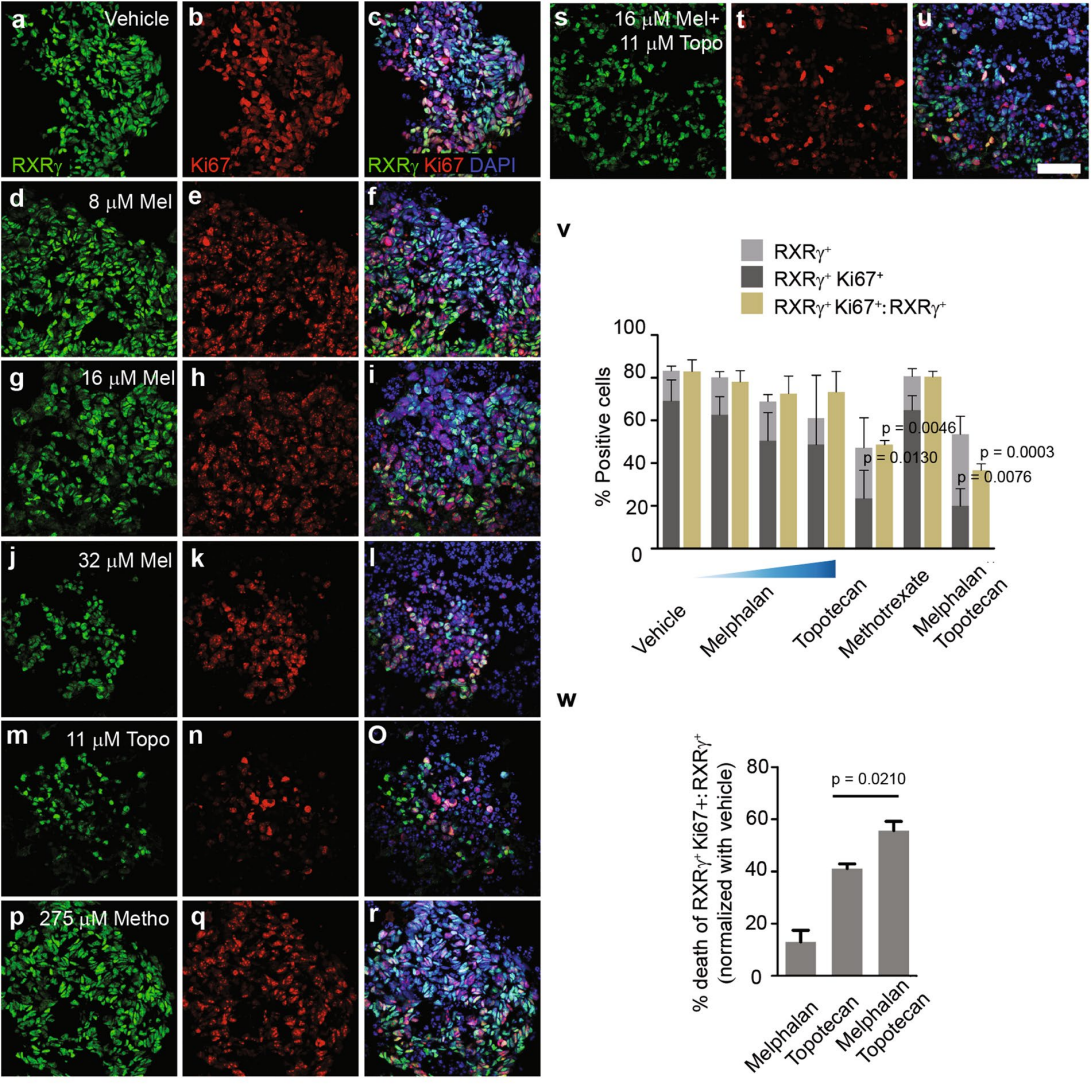
Cone cell features in organoids in response to anticancer drugs at 24 h. (**a–u**) Representative micrographs of co-immunostaining for cone marker RXRγ (**a**,**d**,**g**,**j**,**m**,**p**,**s**) and proliferative marker Ki67 (**b**, **e**,**h**,**k**,**n**,**q**,**t**) in organoids treated with vehicle (**a–c**), 8 (**d–f**), 16 (**g–i**), or 32 (**j–l**) µM melphalan (Mel), 11 µM topotecan (Topo) (**m**–**o**), 275 µM methotrexate (Metho) (**p**–**r**), or the combined regimen of 16 µM melphalan with 11 µM topotecan (**s**–**u**). Merged images (**c**,**f**,**i**,**l**,**o**,**r**,**u**). Nuclei stained by 4′,6-diamidino-2-phenylindole (DAPI). Scale bar, 50 µm. (**v**) Bar graph shows % RXRγ^+^ cells (non-proliferative cones), RXRγ^+^ cells co-stained with Ki67 (proliferative cones), and ratio of proliferative to non-proliferative cones (mean percentages ± SEM, n = 3). Mean percentages (proportions) were determined in nine micrographs for each condition. Statistical analysis of % positive cells was conducted by one-way ANOVA followed by Dunnett’s test. (**w**) Bar graph shows death of cell ratio of RXRγ^+^ Ki67^+^ to RXRγ^+^ (mean percentages ± SEM, n = 3, unpaired t-test).

Anticancer drugs had a genotoxic effect, as shown by elevated γ-H2AX foci in drug-treated organoids (Fig. S7); this ultimately caused cell death (Fig. 5c–i). Although the combined drugs, topotecan and high-dose melphalan, equally induced cell death (Fig. 5j), viable tumor cells that might be capable of regrowth remained in organoids. We asked whether the remaining cells were proliferative tumor cones and which drugs showed rapid control (at 24 h of exposure) by preferentially destroying proliferative cells, rather than resting tumor cone cells. We labeled RXRγ, which is required for the proliferation and survival of RB^17^. Co-expression of RXRγ and Ki67 identified proliferative tumor cone cells and differentiated from RXRγ^+^ Ki67^−^ resting tumor cones (Fig. 6a–u). RXRγ staining indicated that cuboidal or column-shaped cells were maintained as in-vehicle-organoids, suggestive of low efficacy of low and medium doses of melphalan and methotrexate (Fig. 6a,d,g,p). In contrast, organoid cells were transformed into round shapes with the high doses of melphalan, topotecan, and the combined drug regimen (Fig. 6j,m,s). Vehicle-treated organoids consisted of 83.3 ± 2.2% of RXRγ^+^ cells and 69.2 ± 5.7% of RXRγ^+^ Ki67^+^ cells; thus, the cell ratio of RXRγ^+^ Ki67^+^ to RXRγ^+^ was 83.0 ± 5.4% (mean ± SEM) (Fig. 6a–c,v). We found that at 24 h exposure, proportions of viable RXRγ^+^ cells in drug-treated organoids remained as in-vehicle-organoids (Fig. 6a–v). Topotecan and the combined drug regimen both significantly reduced the proportions of viable RXRγ^+^ Ki67^+^ cells (23.5 ± 7.6%, p = 0.0130; 20 ± 4.6%, p = 0.0076) and cell ratios of RXRγ^+^ Ki67^+^ to RXRγ^+^ (48.7 ± 2.0%, p = 0.0046; 36.6 ± 3.1%, p = 0.0003) (Fig. 6m–o,s–v). This suggested that topotecan, both alone and in combination with melphalan, targeted proliferative tumor cones. In comparison with topotecan alone, the combined drug regimen demonstrated an enhanced effect in reducing proliferative tumor cones (the highest single agent model: CI = 0.74) (Fig. 6w).

## Discussion

Culture systems greatly impact the maintenance of tumorigenic aspects of primary tumor-derived cells. Two-dimensional adherent cultures, despite being amenable to high-throughput screening, do not recapitulate and rarely represent clinically relevant patient tissues^40^. The advent of organoid cultures has allowed generation of 3-D, self-organizing cellular structures that resemble tissue. Here, we demonstrated that tumor organoids can be derived from a tumor of the retina and can retain molecular and cellular features of the parental retinal tumor. Organoids had histological structure resembling retinal tumors and seeds. This, in turn, supports the ability of retinal tumors from advanced RB with tumor-associated seeds to generate retinal tumors and seeds in organoid cultures. Additionally, as a model of advanced RB tumor organoids produced different drug responses that can be used to predict anticancer activities of drugs for RB control.

Two subgroups of RB with biallelic loss of the *RB1* gene have been identified; both exhibit gene expression signatures of cone photoreceptors, although the cone-associated genes are expressed more highly in one group than the other^41^. The reduced expression of cone-associated genes is proposed to associate with increased genomic alterations, which contribute to tumor progression^42^. Consistently, RB organoids in our study exhibited well-preserved cone gene expression signatures and cone-specific proteins, reflective of the tumor cell of origin^17,18^. In addition, enriched expression of genes associated with mesenchymal cell and fibroblast proliferation in organoids implies that epithelial-mesenchymal transition is induced in tumor cells for invasiveness^43–45^, consistent with the characteristics of primary RB that invaded choroid or optic nerve. We detected additional regional gains in organoids, which could represent undetectable genomic disruptions within the original tumors and may coevolve through a Darwinian selection process to increase the fitness of the overall tumor population^46^. These alterations, such as regional 2p gain, have been documented in primary RB^37,38^ and allow the emergence of a complex clonal architecture that may underlie tumor proliferation, progression, or drug resistance.

Analysis of RB1-depleted retinal cells identifies differentiating cones as tumor-initiating cells that form RB-like tumors in orthotopic xenografts^18^. Human cone-specific signaling circuitry sensitizes to cancerous transformation and collaborates with RB1 depletion^17,18^. An intrinsically high level of expression of the MDM2 proto-oncogene in human cones predisposes them to transformation by preventing cell death^17,47^. MDM2 expression is regulated by the cone-specific RXRγ^17,47^, which, together with TRβ2^17,48^, is required for the proliferation and survival of RB. The expression of MDM2 is not detected in xenografts^12^, but this gene was expressed in our tumor organoids, together with RXRγ and TRβ2. This indicates that organoids retain cone-specific signaling circuitry, suggesting the use of tumor organoids as a model for examining targeted therapies specifically designed to destroy this circuitry.

Organoids provide opportunities for testing the accessibility of therapeutic agents and *ex vivo* screening of drug sensitivities. We found that combined treatment with topotecan and melphalan was more effective than melphalan alone, consistent with clinical outcomes observed in attempts to control vitreous seeds^5,11^. Melphalan (20–30 µg) is extensively used in intravitreal chemotherapy, but in some cases fails to control recurrent and refractory seeds^3,5^. The combined drug regimen achieves rapid control of seeds, such that fewer cycles of chemotherapy are required, compared with melphalan alone^5,11^. Because of its limited toxicity^49^, topotecan alone has been recently used to manage persistent vitreous seeds with satisfactory outcomes^6^; its efficacy is between that of melphalan alone and the combined drug regimen^5,6,11^, consistent with our results. Partial control of seeds has been achieved with low-dose melphalan (8–10 µg), consistent with our results, while higher doses of melphalan (>40 µg) cause ocular complications^4^. Unlike other drugs, methotrexate had slow effects and exhibited the lowest efficacy, consistent with the need for multiple injections over a longer period of treatment^10^. Topotecan alone and in combination with melphalan effectively targeted proliferative cones, rather than non-proliferative cones. Topotecan, a topoisomerase I inhibitor, induces rapid cellular stress in G1, G2, and S phases, thereby causing failure to engage mitosis^50^. We routinely use melphalan and methotrexate with variable success in controlling vitreous seeds. The results of the current study are consistent with previous reports^6,11^ that encouraged the use of topotecan and melphalan in management of vitreous seeds. In addition, these two drugs through intravitreal chemotherapy demonstrate therapeutic effects against subretinal seeds or recurrent retinal tumors^8,9^.

The tumor microenvironment, or tumor stroma, is highly responsible for growth, metastasis, and drug resistance through paracrine effects^51,52^. Glial cells with astrocyte properties, which serve as the tumor microenvironment, promote proliferation and survival of RB^53^. Organoids and tumor tissue contained glial cells, as indicated by GFAP^+^ cell staining, which constitute ∼2–3% of the cells in RB tumors^17^. The expression of GDNF (by glia or fibroblasts), and its cognate receptor RET, in organoids and the parental tumor (Fig. S5b,c) implies crosstalk between glia and tumor cells. Unlike in tumor organoids, GFAP^+^ glial tumor microenvironment is absent in tumorspheres derived from RB tissues^40^. In comparison with tumorsphere culture^40^, the key difference is the utilization of extracellular matrix (Matrigel®) in organoid culture for embedding tumor cells. Laminin, a major component in matrix, promotes the formation of 3-D cellular structure and histogenesis of epithelial organoids^54,55^, thus creating the features that are not found in tumorspheres^40^.

In the era of precision medicine, faithful preclinical models are important for guiding treatment options. Organoid technology offers simple and efficient generation of 3-D-tumor models. RB organoid models retained cone signal circuitry and produced clinically relevant drug responses, thus facilitate development of targeted therapies that can be used in management of advanced RB. As a model, organoids could accelerate the discovery of novel therapies, while reducing animal usage and costs invested in therapeutic development.

## Materials and Methods

### Human tissues

RB tissues were collected directly from patients undergoing enucleation. Tumor tissue samples after incision were used for organoid culture and for analyses of DNA copy number alterations and gene expression profiles. Tumors in enucleated globes were examined by ophthalmologist; fresh surgical specimens were obtained and collected in ice-cold collecting medium (Dulbecco’s Modified Eagle Medium: Nutrient Mixture F-12 (DMEM: F-12) containing 0.25 μg/mL amphotericin B and 100 U/mL penicillin-streptomycin). Surgical specimens were processed for culture within 1 h after the tumor tissues were incised from enucleated globes; the globes were then fixed for pathological analysis. Blood was drawn from patients for analysis of DNA copy number. All experimental protocols were approved by the IRB at Faculty of Medicine, Ramathibodi Hospital, Mahidol University (protocol number ID11–58–53 and ID07-60-14). All methods were performed in accordance with the relevant guidelines and regulations. Informed consent was obtained from a parent of each patient before the samples were collected.

### RB organoid culture

Tumor tissues were finely minced and incubated in ACCUMAX™ (Chemicon) for 30 min at 37 °C. One volume of PBS was added to the cell solution, which was then centrifuged at 300 × *g* for 5 min. Supernatant was removed and cell pellets were resuspended in cold organoid medium (Neurobasal medium (Invitrogen) supplemented with 20 pg/mL EGF (R&D Systems), 10 pg/mL bFGF (Peprotech), 1X B27 (Invitrogen), 2.5% knockout serum replacement, 2.5% fetal bovine serum, 20 mM Glutamax, 1 mM sodium pyruvate, 0.25 µg/mL amphotericin B, and 100 U/mL penicillin-streptomycin). Tumor cell solution was embedded in Matrigel® (growth factor reduced, Corning) at a 1:1.8 ratio of cell solution to Matrigel® solution. A total of 20 µL mixed cell-gel solution was added to six-well plates via 5–7 drops/well and solidified in an incubator (37 °C) for 30–45 min. Organoid medium was added to cover the gel drops; cultures were maintained in a humidified incubator, with 5% CO_2_, at 37 °C. RB organoids were manually dissociated and passaged at a 1:3 or 1:4 ratio every 3–4 weeks by embedding in fresh Matrigel®. Cold freezing medium (organoid medium containing 10% dimethylsulfoxide) was used to freeze organoids at −80 °C for 24 h prior to long-term storage in liquid nitrogen.

### Drug treatments

Drugs (pharmaceutical grade) were further diluted with 0.9% NaCl to obtain concentrations equivalent to the final clinical dose achieved in the vitreous, including melphalan at 8 (10), 16 (20) and 32 (40) µM (µg of delivered drugs in vitreous-containing 4 mL fluid), methotrexate at 275 µM (400 µg), and a combination of melphalan at 16 µM (20 µg) and topotecan at 11 µM (30 µg). Organoids (<passage 5) were incubated with drugs for 24 or 72 h. NaCl (0.02% final concentration in culture) was used as a control.

Histology, immunofluorescence and imaging, cell cycle, copy number, and gene expression analyses are described in the Supplementary Information.

## Electronic supplementary material

Supplementary Information

## Data Availability

All data generated or analyzed during this study are included in this published article and its Supplementary Information files. RNA-seq data have been deposited in Gene Expression Omnibus (GEO) through accession number GSE120710 (www.ncbi.nlm.nih.gov/geo/query/acc.cgi?acc=GSE120710).
