# Supplementary material for: A three-dimensional organoid model recapitulates tumorigenic aspects and drug responses of advanced human retinoblastoma
Source: Sci Rep. 2018 Oct 23;8:15664. doi: 10.1038/s41598-018-34037-y (PMC6199308; doi:10.1038/s41598-018-34037-y)
Supplement: Supplementary file 1 — Supplementary Information [file 41598_2018_34037_MOESM1_ESM.pdf]

A three-dimensional organoid model recapitulates tumorigenic aspects and drug responses of advanced human retinoblastoma

Duangporn Saengwimol[1], Duangnate Rojanaporn[2], Vijender Chaitankar[3], Pamorn Chittavanich[4], Rangsima Aroonroch[5], Tatpong Boontawon[4], Weerin Thammachote[4], Natini Jinawath[4], Suradej Hongeng[6] and Rossukon Kaewkhaw[*, 4]

[1]Research Center, [2]Department of Ophthalmology, [4]Section for Translation Medicine, [5]Department of Pathology and [6]Department of Pediatrics, Faculty of Medicine Ramathibodi Hospital, Mahidol University, Bangkok, Thailand. [3]Bioinformatics Computational Biology Core, National Heart, Lung, and Blood Institute (NHLBI), National Institutes of Health (NIH), Bethesda, USA.

[*]Corresponding author: Rossukon Kaewkhaw, Mahidol University, Faculty of Medicine Ramathibodi Hospital, Section for Translational Medicine, Bangkok, 10400, Thailand. Phone: 66-201-2615; Fax: 66-201-02971137; E-mail: rossukon.kae@mahidol.ac.th.

Supplementary Materials and Methods

Supplementary Figures

Supplementary Materials and Methods

Histology, immunofluorescence and imaging

Tumor organoids were fixed with 4% paraformaldehyde solution for 15 min, washed with phosphate-buffered saline (PBS) and incubated in 30% (w/v) sucrose overnight, then embedded in OCT compound and snap frozen. Cryosections (10 µm) of tumor organoids were mounted on SuperFrost Plus slides for immunostaining. Retinoblastoma (RB) tissue/organoids were fixed, dehydrated, and embedded in paraffin. Paraffin sections (4 µm) were stained by hematoxylin and eosin for histological analysis. Cryosections of organoids or paraffin sections of RB tissue were stained with primary (overnight) and secondary antibodies. Paraffin sections were deparaffinized and rehydrated, then antigen retrieval was performed by heating at 95–100°C in buffer (10 mM sodium citrate, 0.05% Tween 20, pH 6.0) for 15 min prior to staining.

The following antibodies were used for staining: AP-2 alpha (1:35, mouse, 3B5, DSHB), BRN-3 (1:200, goat, sc-6026), CHX10 (1:200, goat, sc-21690), RXR gamma (1:100, mouse, sc-365252) from Santa Cruz Biotechnology; GFAP (1:50, mouse, G3893, Sigma); H2AX gamma (1: 400, rabbit, 9718S, Cell Signaling); Ki67 (1:100, mouse, 550609, BD Pharmingen™); Ki67 (1:100, rabbit, RB1510P0, Thermo Scientific™ Lab Vision™); M/L opsin and S-opsin (1:5000, rabbit, gift of Dr. Nathan); NRL (1:1000, mouse, gift of Dr. Swaroop); PAX6 (1:400, mouse, PAX6, DSHB); PROX1 (1:1000, rabbit, AB5475, Millipore); Rhodopsin (1:100, mouse, Rho4D2, Gift of Dr. Molday); cleaved caspase3 (1:400, rabbit, 9661, Cell Signaling); phospho-Histone H3 (1:150, rabbit, 9701, Cell Signaling); and TRβ2 (1:100, rabbit, Gift of Dr. Forrest). Secondary antibodies (1:500) included Alexa Fluor 555 Donkey anti-Goat IgG, Alexa Fluor 568 Goat anti-Rabbit IgG, Alexa Fluor 568 Donkey anti-Mouse IgG, Alexa Fluor 488 Goat anti-Rabbit IgG and Alexa Fluor 488 Goat anti-Mouse IgG (Invitrogen). Phalloidin (1:100, Invitrogen) was used for actin staining. Nuclei were counterstained by 4',6-diamidino-2-phenylindole (DAPI). Fluorescent images were acquired by confocal laser scanning

microscopy and Z-stacking was performed with NIS-Element AR (Nikon). Mosaic bright-field images of organoids were captured by Axiovert A1 and assembled by ZEN lite (Carl Zeiss).

Cell cycle analysis

Tumor organoids were dissociated to yield single cells by TrypLE and fixed in cold 70% ethanol for 2 h at -20°C. Organoid cells were stained with propidium iodine (PI) staining solution (50 μg/ml, with 100 μg/ml RNase and 2 mM $MgCl_2$ in PBS) for 30 min before analysis of DNA contents by flow cytometry. Data were acquired by using a BD FACSVerse system set at 10,000 events.

Copy number analysis

DNA was extracted from primary tumor tissues, organoids, or blood by using DNeasy Blood & Tissue Kits (Qiagen). The quality of DNA specimens was confirmed by agarose gel electrophoresis and the concentration was measured with the Qubit dsDNA BR Assay. *RB1* mutations were screened in tumor tissue, organoids, and blood by using direct PCR sequencing. Two hundred nanograms of DNA were used for analyses of copy number alterations and loss of heterozygosity, by using the CGH/SNP array (Infinium CytoSNP-850K array, Illumina) in accordance with the manufacturer's instructions; the results were analyzed and visualized with copy number software (Nexus, BioDiscovery).

RNA sequencing

RNA was extracted from primary RB and tumor organoids at P1 and P3 (6 and 13 weeks post-establishment) by using TriPure isolation reagent (Roche Applied Science). The RNA quality and quantity were determined by RNA6000 assay (Agilent). Specimens with an RNA Integrity Number (RIN) > 8.0 were used in this study. RNA Libraries were constructed by using the TruSeq Stranded mRNA LT Sample Prep Kit (Illumina), in accordance with the manufacturer's

instructions. RNA sequencing was performed with the Illumina NavaSeq sequencing system (100-bp paired-end reads); an average of 70 million reads were generated for each sample.

RNA-seq quantification and differential expression analysis

High-quality RNA-seq reads were selected by using Trimmomatic (v0.36). Kallisto (v0.43) was used to compute transcript-level counts on the Ensembl V84 transcriptome (coding and non-coding sequences were included to compute index with Kallisto). To compute gene level expression, the “tximport” R package was used. Initially, gene level count data was TMM (trimmed mean of M-values); it was normalized and then underwent CPM (counts per million) computation. To account for differences in sequencing, we modeled the data with two batches representing poly-A-tail-pulldown vs. total RNA sequencing protocols. We incorporated these batches in our design matrix and implemented a generalized linear modeling approach for differential expression (“estimateDisp,” followed by “glmFit” functions in the “edgeR” package). Genes with $\log_2$ fold-change of 2 and Benjamini-Hochberg-adjusted p-values ≤ 0.01 were selected as differentially expressed genes.

Gene-ontology enrichment analysis

Gene ontology (GO) analysis of differentially expressed genes was performed by using the “enrichGO” function in the “clusterProfiler” R package (v3.6.0) (PMID: 22455463); the list of all expressed genes (≥ 1 CPM in samples of organoid, RB, or normal samples) was used as the universe in the analysis function.

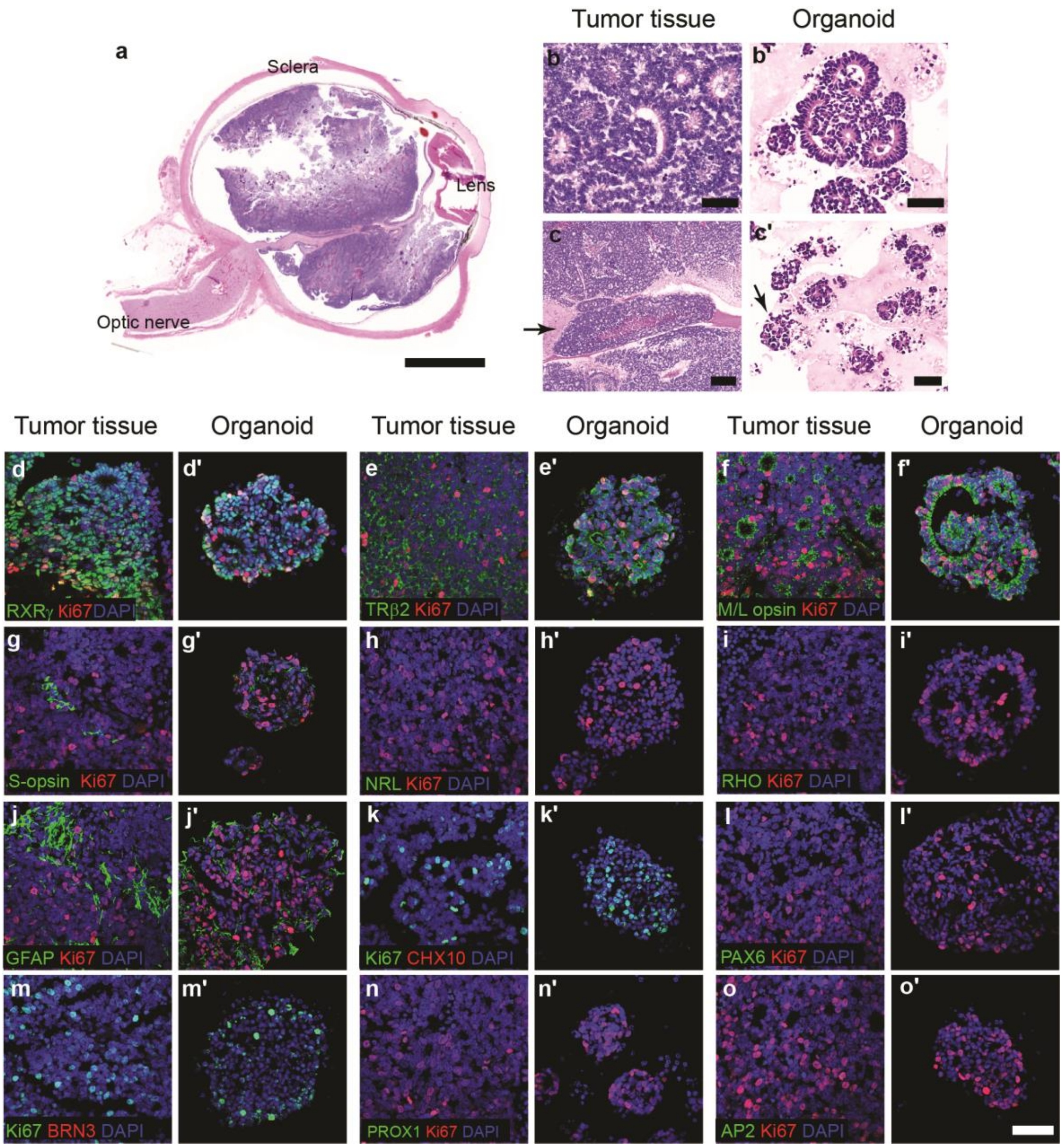

Supplementary Figure 1. Reproducible cellular features and contents of the retinoblastoma in tumor organoids (RB654)

(**a**) Hematoxylin and eosin staining of the enucleated globe. (**b–c’**) Representative micrographs indicate histological features of tumor tissue and organoids. The presence of Flexner-Wintersteiner rosettes, indicative of well-differentiated retinal tumor (b), and vitreous seed, a cell cluster with an outer rim of viable cells surrounding central necrotic/apoptotic cells (arrow) (c), in tissue. The corresponding structures are identified in organoid cultures: rosettes (b’) and vitreous seed-like features, a small spherical clusters of viable cells (arrow) (c’).
(**d–o’**) Representative micrographs of co-immunostaining indicate the expression of Ki67 and retinal proteins [RXRγ (d, d’), TRβ2 (e, e’) and M/L opsin (f, f’), S- opsin (g, g’), NRL (h, h’), RHO (i, i’), GFAP (j, j’), CHX10 (k, k’), PAX6 (l, l’), BRN3 (m, m’), PROX1 (n, n’) and AP2 (o, o’)] in parental tumor tissue and organoids. Nuclei stained by 4',6-diamidino-2-phenylindole (DAPI). Scale bar, 5 mm (a); 50 µm (b, b’, c’); 100 µm (c) and 50 µm (d–o’).

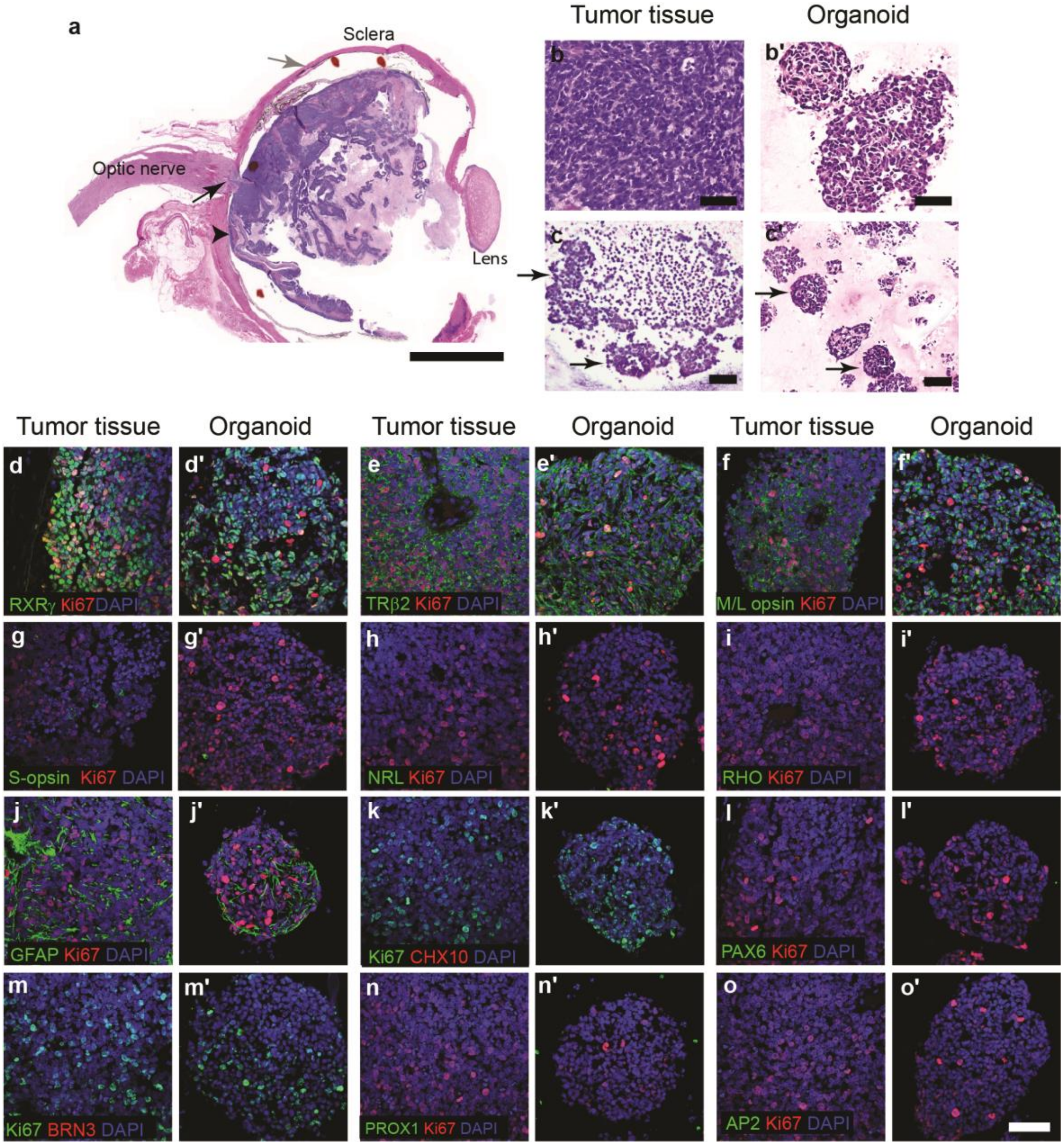

Supplementary Figure 2. Reproducible cellular features and contents of the retinoblastoma in tumor organoids (RB187)

(**a**) Hematoxylin and eosin staining of the enucleated globe (optic nerve invasion at prelaminar area (black arrow); chroroidal invasion (gray arrow) and subretinal seed (arrow head)). (**b–c'**) Representative micrographs indicate histological features of tumor tissue and organoids. The presence of poorly differentiated retinal tumor (b) and vitreous seed, small spherical clusters of viable cells (arrow) (c), in tumor tissue. The corresponding structures are identified in organoids, indicating poorly differentiated features of tumor (b') and vitreous seed-like features (c'). (**d–o'**) Representative micrographs of co-immunostaining indicate the expression of Ki67 and retinal proteins [RXRγ (d, d'), TRβ2 (e, e') and M/L opsin (f, f'), S- opsin (g, g'), NRL (h, h'), RHO (i, i'), GFAP (j, j'), CHX10 (k, k'), PAX6 (l, l'), BRN3 (m, m'), PROX1 (n, n') and AP2 (o, o')] in parental tumor tissue and organoids. Nuclei stained by 4',6-diamidino-2-phenylindole (DAPI). Scale bar, 5 mm (a); and 50 µm (b–o').

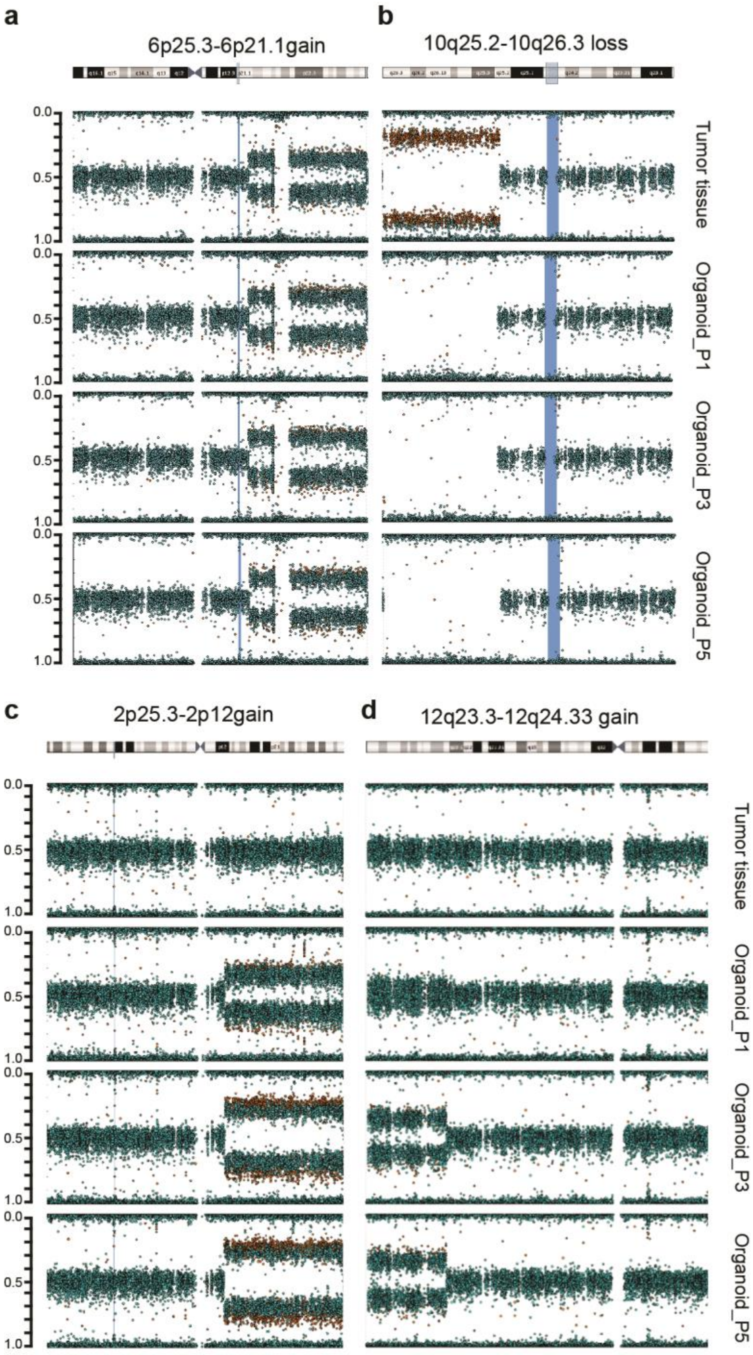

Supplementary Figure 3. Regional recurrent gain or loss found in tumor organoids and tissue (RB668)

(**a–b**) B-allele frequency indicates mosaicism of recurrent somatic copy number variation found in tumor organoids at passage 1 (P1, 6-week culture), 3 (P3, 13-week culture), 5 (P5, 19-week culture) and tissue. The sub-clonal neoplastic cells with regional gain at 6p (a) and/or loss at 10q (b) were enriched in tumor organoids. (**c–d**) B-allele frequency indicates mosaicism of gains at 2p (c) and 12q (d) in organoids. The sub-clonal population with 2p and/or 12q gains increased with additional passaging of organoids. Vertical blue line represents loss of heterozygosity (LOH).

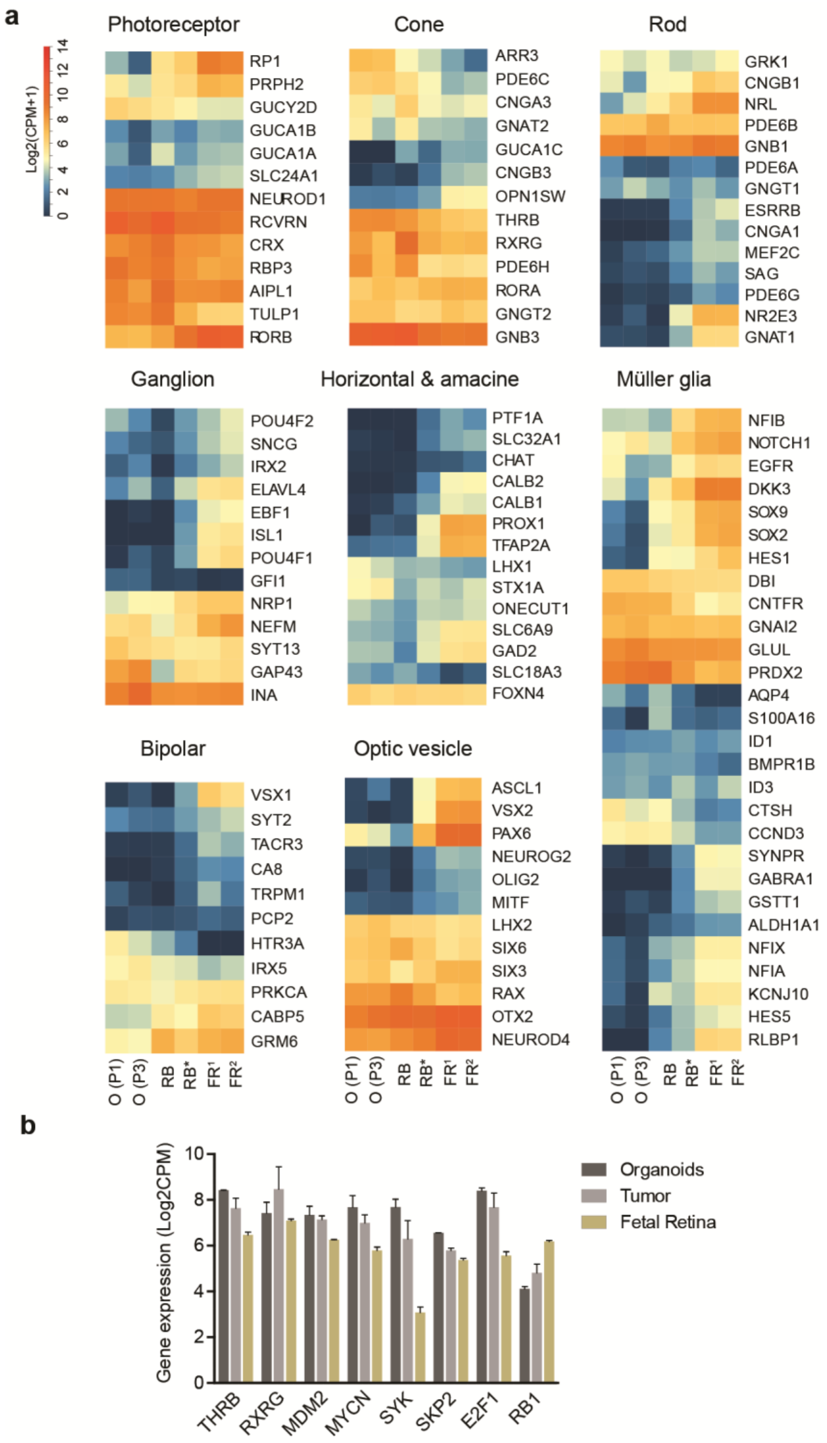

Supplementary Figure 4. Gene signatures representing different retinal cell types

(**a**) Heat maps show gene expression profiles of organoids (O) at passage 1 (P1, 6-week culture) and 3 (P3, 13-week culture), the corresponding patient-derived retinoblastoma (RB) and published transcriptomes of retinoblastoma (RB*) and fetal retina (FR[1] and FR[2]). (**b**) Gene expression of cone signal circuitry susceptible to RB transformation in organoids, tumor and fetal retina.

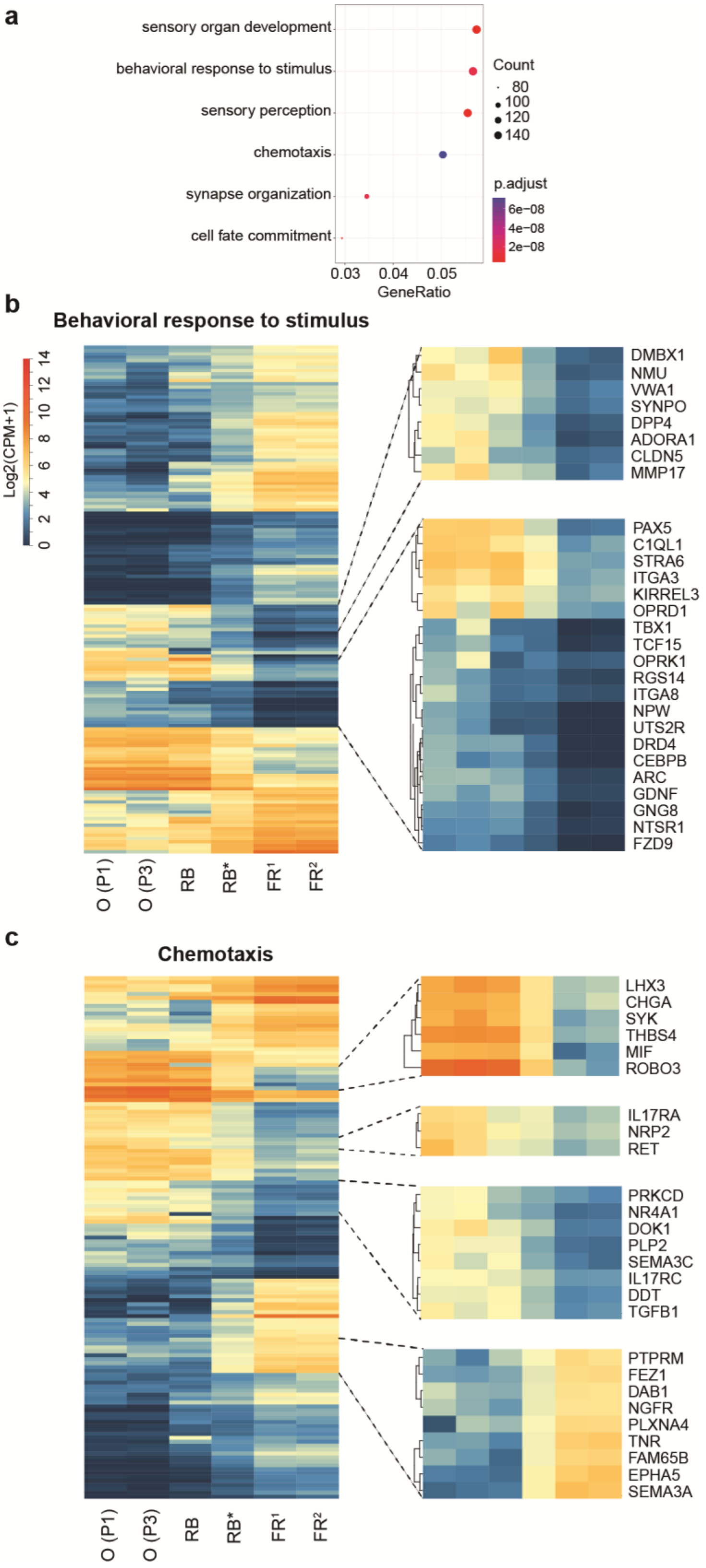
a
sensory organ development
behavioral response to stimulus
sensory perception
chemotaxis
synapse organization
cell fate commitment
0.03
0.04
0.05
GeneRatio
Count
80
100
120
140
p.adjust
6e−08
4e−08
2e−08
b
Behavioral response to stimulus
Log2(CPM+1)
0
2
4
6
8
10
12
14
DMBX1
NMU
VWA1
SYNPO
DPP4
ADORA1
CLDN5
MMP17
PAX5
C1QL1
STRA6
ITGA3
KIRREL3
OPRD1
TBX1
TCF15
OPRK1
RGS14
ITGA8
NPW
UTS2R
DRD4
CEBPB
ARC
GDNF
GNG8
NTSR1
FZD9
O (P1)
O (P3)
RB
RB*
FR1
FR2
c
Chemotaxis
LHX3
CHGA
SYK
THBS4
MIF
ROBO3
IL17RA
NRP2
RET
PRKCD
NR4A1
DOK1
PLP2
SEMA3C
IL17RC
DDT
TGFB1
PTPRM
FEZ1
DAB1
NGFR
PLXNA4
TNR
FAM65B
EPHA5
SEMA3A
O (P1)
O (P3)
RB
RB*
FR1
FR2

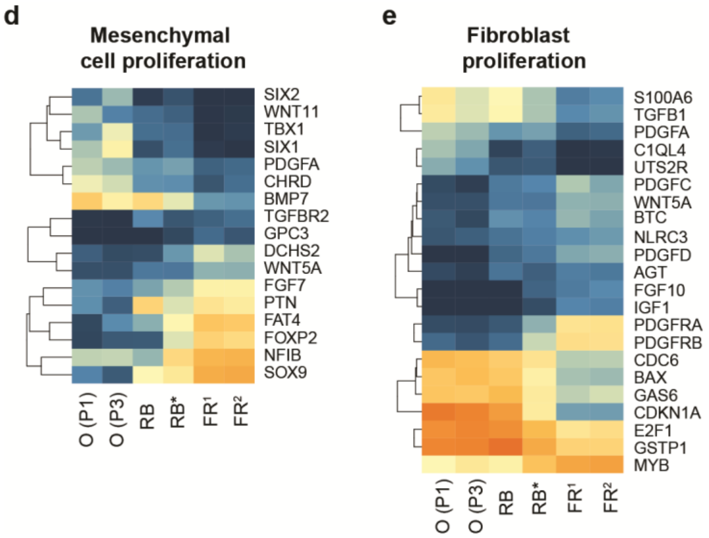

Supplementary Figure 5. Gene ontology (GO) and differentially expressed genes associated with GO terms

(**a**) Top six significantly enriched GO terms obtained in a comparison of differentially expressed genes between organoids and fetal retina. (**b–e**) Heat maps show differentially expressed genes associated with GO terms: behavioral response to stimulus (b), chemotaxis (c), mesenchymal cell proliferation (d) and fibroblast proliferation (e). Abbreviations: O, organoid (RB668); RB, the corresponding patient-derived retinoblastoma; RB*, published transcriptomes of retinoblastoma; FR, fetal retina; and P, passage.

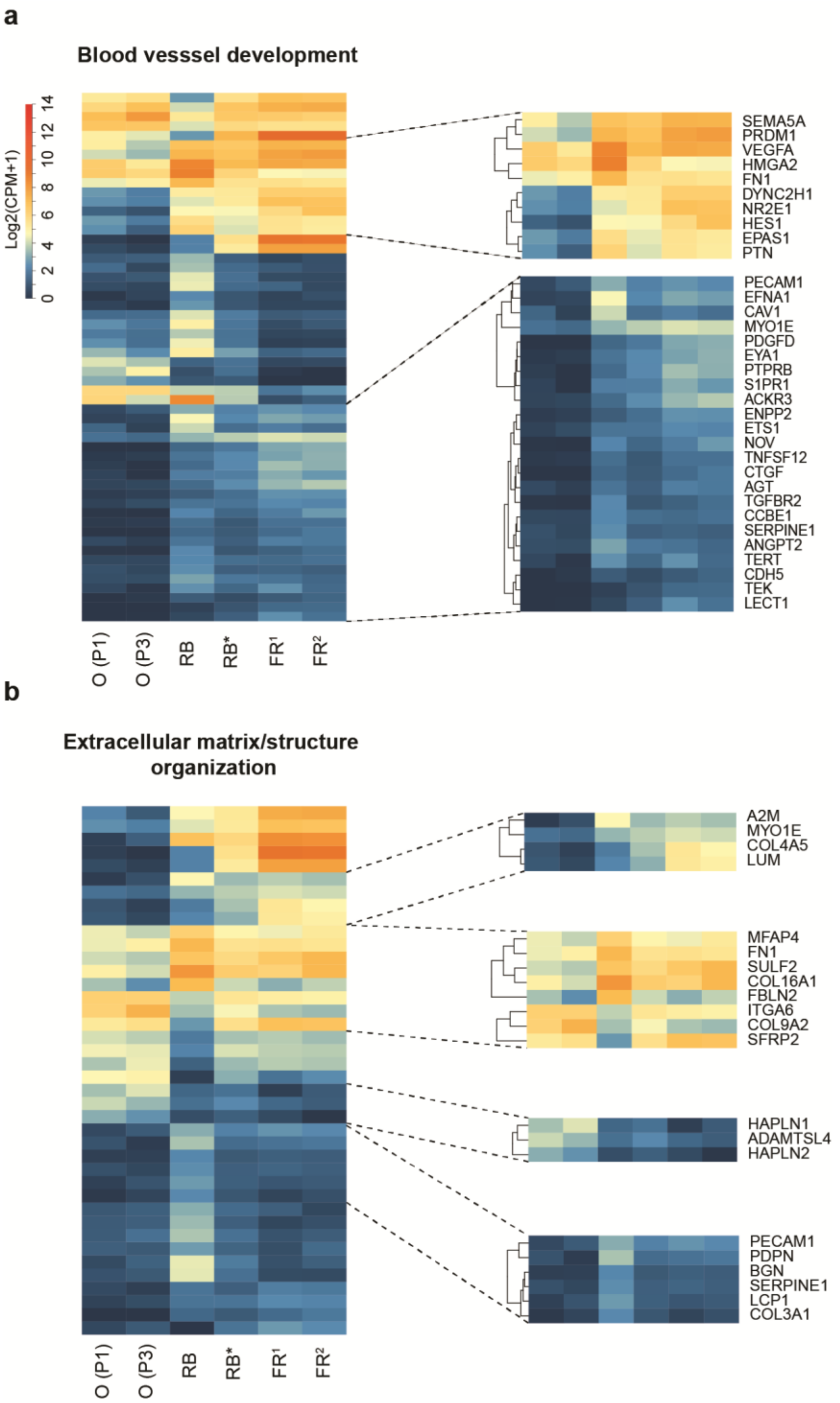

Supplementary Figure 6. Gene Ontology (GO) and differentially expressed genes in organoids and retinoblastoma tissue

(a, b) Heat maps show differentially expressed genes associated with the significantly enriched GO terms: blood vessel development (a) and extracellular matrix/structure organization (b). Abbreviations: O, organoid (RB668); RB, the corresponding patient-derived retinoblastoma; RB*, published transcriptomes of retinoblastoma; FR, fetal retina; and P, passage.

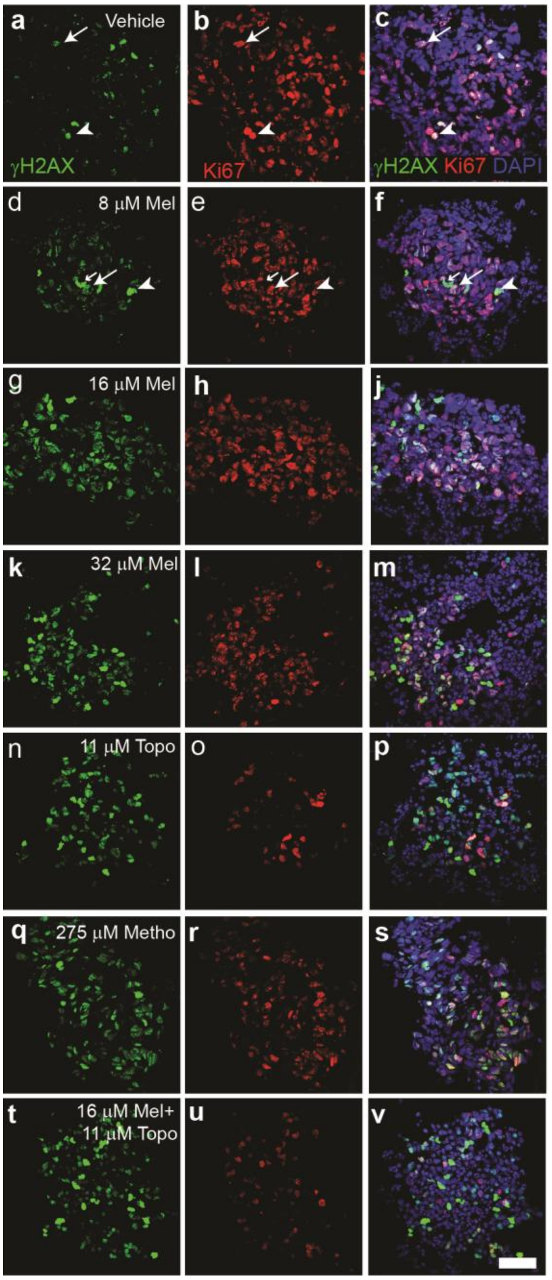

Supplementary Figure 7. DNA damage response after drug administration

(**a–v**) Representative micrographs of immunostaining for a marker of DNA damage response γH2AX foci (a, d, g, k, n, q, and t) indicate drug accessibility to the core or organoids at 24 h after treatment. Sections were co-labeled with Ki67 (b, e, h l, o, r, and u). Merged images (c, f, j, m, p, s, and v). Organoids treated with vehicle (a–c), 8 (d–f), 16 (g–j), and 32 (k–m) µM melphalan (Mel) , 11 µM topotecan (Topo) (n–p), 275 µM methotrexate (Metho) (q–s) and combined 16 µM melphalan with 11 µM topotecan (t–v). Large arrows indicate organoid cells forming γH2AX foci. Arrowheads (γH2AX^{+} Ki67^{+}) indicate organoid cells undergoing mitotic γH2AX phosphorylation, whereas small arrows (γH2AX^{+} Ki67^{-}) indicate apoptotic cells. Nuclei stained by 4',6-diamidino-2-phenylindole (DAPI). Scale bar, 50 µm.
